# ACCELERATING DEMENTIA RESEARCH AND EARLY-CAREER DEVELOPMENT THROUGH THE SUMMER RESEARCH INSTITUTE

**DOI:** 10.1093/geroni/igad104.2543

**Published:** 2023-12-21

**Authors:** Chelsea Kline, Matthew Baumgart, Julie Bynum, Sheryl Zimmerman, Sam Fazio

**Affiliations:** Alzheimer’s Association, Chicago, Illinois, United States; Alzheimer’s Association, New York, New York, United States; University of Michigan, Ann Arbor, Michigan, United States; University of North Carolina at Chapel Hill, Chapel Hill, North Carolina, United States; Alzheimer’s Association, Chicago, Illinois, United States

## Abstract

Today, there are more than six million Americans living with Alzheimer’s disease and more than 11 million unpaid caregivers. By 2060, the number of Americans living with Alzheimer’s could reach 14 million. These statistics highlight the need for early career investigators to join the dementia research workforce and accelerate breakthroughs in this field. In 2020, the National Institutes on Aging (NIA) funded the Alzheimer’s Association Interdisciplinary Summer Research Institute (AA-ISRI) focused on early career investigators interested in psychosocial and public health research related to dementia. AA-ISRI is a five-day immersive experience in which 24 early career investigators are selected to come to Chicago, attend didactic lectures led by expert faculty, develop research proposals in small groups, and receive one-to-one mentoring. Participants leave AA-ISRI with a greater understanding of the tools needed to succeed in dementia research and an extensive network of colleagues and mentors. Based on evaluations collected from participants from the first two years of the program, 97% of respondents indicated they were satisfied with the program, 94% felt the program increased their understanding of Alzheimer’s, and 90% said the program improved their independent research skills. Participants have shared that AA-ISRI is a highly unique opportunity that every early career investigator in the field should attend. Alumni from the first two years of AA-ISRI have gone on to publish 40+ peer-reviewed publications and have been awarded 15+ grants. This poster will review the development of AA-ISRI, key outcomes, and its impact on the future of dementia research.

